# Effects of chronic secondhand smoke exposure on cardiovascular regulation and the role of soluble epoxide hydrolase in mice

**DOI:** 10.3389/fphys.2023.1185744

**Published:** 2023-06-08

**Authors:** Shiyue Pan, Emma Karey, Madeline Nieves-Cintron, Yi-Je Chen, Sung Hee Hwang, Bruce D. Hammock, Kent E. Pinkerton, Chao-Yin Chen

**Affiliations:** ^1^ Department of Pharmacology, University of California Davis, Davis, CA, United States; ^2^ Department of Entomology and Nematology, UC Davis Comprehensive Cancer Center, University of California Davis, Davis, CA, United States; ^3^ Center for Health and the Environment, University of California Davis, Davis, CA, United States

**Keywords:** sidestream smoke, autonomic function, baroreflex sensitivity, cardiovascular effect, heart rate variability, soluble epoxide hydrolase

## Abstract

**Background:** Secondhand smoke (SHS) is a significant risk factor for cardiovascular morbidity and mortality with an estimated 80% of SHS-related deaths attributed to cardiovascular causes. Public health measures and smoking bans have been successful both in reducing SHS exposure and improving cardiovascular outcomes in non-smokers. Soluble epoxide hydrolase (sEH) inhibitors have been shown to attenuate tobacco exposure-induced lung inflammatory responses, making them a promising target for mitigating SHS exposure-induced cardiovascular outcomes.

**Objectives:** The objectives of this study were to determine 1) effects of environmentally relevant SHS exposure on cardiac autonomic function and blood pressure (BP) regulation and 2) whether prophylactic administration of an sEH inhibitor (TPPU) can reduce the adverse cardiovascular effects of SHS exposure.

**Methods:** Male C57BL/6J mice (11 weeks old) implanted with BP/electrocardiogram (ECG) telemetry devices were exposed to filtered air or 3 mg/m^3^ of SHS (6 hr/d, 5 d/wk) for 12 weeks, followed by 4 weeks of recovery in filtered air. Some mice received TPPU in drinking water (15 mg/L) throughout SHS exposure. BP, heart rate (HR), HR variability (HRV), baroreflex sensitivity (BRS), and BP variability were determined monthly.

**Results:** SHS exposure significantly decreased 1) short-term HRV by ∼20% (*p* < 0.05) within 4 weeks; 2) overall HRV with maximum effect at 12 weeks (−15%, *p* < 0.05); 3) pulse pressure (−8%, *p* < 0.05) as early as week 4; and 4) BRS with maximum effect at 12 weeks (−11%, *p* < 0.05). Four weeks of recovery following 12 weeks of SHS ameliorated all SHS-induced cardiovascular detriments. Importantly, mice exposed to TPPU in drinking water during SHS-related exposure were protected from SHS cardiovascular consequences.

**Discussion:** The data suggest that 1) environmental relevant SHS exposure significantly alters cardiac autonomic function and BP regulation; 2) cardiovascular consequences from SHS can be reversed by discontinuing SHS exposure; and 3) inhibiting sEH can prevent SHS-induced cardiovascular consequences.

## 1 Introduction

Secondhand smoke (SHS), a major indoor air pollutant, consists of 80%–85% sidestream smoke from the burning cigarette and 15%–20% mainstream smoke exhaled from the smoker (Penn et al., 1994; Zhang et al., 2001). While SHS exposure decreased by seven percent points between 2009 and 2018, the prevalence of SHS exposure remained higher in some demographic groups, including non-Hispanic black (40%), family income below the federal poverty level (35%), and younger adults (26% for adults aged 18-39) (Brody et al., 2021). About 80% of SHS-related deaths are due to cardiovascular causes in adults over 20 (Max et al., 2012) suggesting that the cardiovascular system is extremely vulnerable to SHS. Smoking bans that have reduced SHS exposure in public places have been associated with improved cardiovascular outcomes in non-smokers (Meyers et al., 2009; Kelleher and Frazer, 2014).

Altered autonomic function, as indexed by decreased heart rate variability (HRV), is a well-established risk factor for cardiac events including arrhythmias and sudden cardiac death (Villareal et al., 2002) and an important role in SHS exposure-induced acute cardiac consequences (Pope et al., 2001; Raupach et al., 2006; Zhang et al., 2013). In humans, 2 hours of SHS exposure (up to 0.15 mg/m^3^) in a public airport was sufficient to attenuate HRV during the exposure period (Pope et al., 2001); HRV recovered after the subjects were removed from smoking areas (Pope et al., 2001). Previously published work from our laboratory found that 3 days of exposure to a high concentration of SHS (30 mg/m^3^) reduced HRV and increased arrhythmia susceptibility 24 h post-exposure in mice (Chen et al., 2008). However, 3 days of exposure to a more environmentally relevant concentration of SHS (3 mg/m^3^) was not sufficient to produce a sustained effect on HRV beyond the exposure period (Chen et al., 2008). Thus, the first goal of this study was to determine whether a more prolonged environmentally relevant SHS exposure induces changes in autonomic function beyond the exposure period using telemetry recordings in conscious, freely moving mice.

Chronic exposure to SHS contributes to the development of cardiovascular and cardiometabolic diseases, such as hypertension, atherosclerosis, coronary heart diseases, insulin resistance, and diabetes (Glantz and Parmley, 1991; Raupach et al., 2006). Epidemiological studies consistently demonstrated strong associations between SHS exposure and hypertension prevalence, however, the effect of SHS on blood pressure (BP) is often trivial (2–4 mmHg increase in BP) (Wu et al., 2017; Park et al., 2018; Tamura et al., 2018; Kim et al., 2021). While high BP is a well-known risk factor for cardiovascular morbidity and mortality, altered baroreflex function and increased BP variability (BPV) have been shown to cause more cardiovascular end-organ damage than elevated BP (Lanfranchi and Somers, 2002; Stevens et al., 2016). However, the impact of SHS on baroreflex function and BPV are not well characterized. Thus, the second goal of this study was to characterize the effect of SHS on BP regulation.

At the point of entry, cigarette smoke activates macrophage release of proinflammatory cytokines, leading to the recruitment of inflammatory cells into the lung where they subsequently release inflammatory mediators and deplete protective antioxidants (Li et al., 1994; Gardi and Valacchi, 2012). Soluble epoxide hydrolase (sEH) catalyzes the metabolism of anti-inflammatory epoxides such as EETs to pro-inflammatory diols such as dihydroxyeicosatrienoic acids (DHETs) (Yang et al., 2011; Pillarisetti and Khanna, 2012; Wang et al., 2013). Stabilizing EETs through the inhibition sEH is an effective approach to reduce and resolve inflammation (Ingraham et al., 2011; Qiu et al., 2011). Of relevance, sEH inhibitors have been shown to significantly attenuate acute tobacco exposure-induced increase in macrophages, neutrophils, and lymphocytes in the lung bronchoalveolar fluid (Smith et al., 2005; Nording et al., 2015). In a sub-chronic tobacco smoke exposure study (4 weeks), sEH inhibitors significantly inhibited exposure-induced lung inflammatory responses, respiratory resistance, tissue damping, and vascular remodeling (Wang et al., 2012). Thus, the third goal of this study was to determine whether sEH inhibition can attenuate SHS-induced autonomic dysfunction and cardiovascular dysregulation.

## 2 Materials and methods

All protocols were approved by the University of California, Davis Institutional Animal Care and Use Committee in compliance with the Animal Welfare Act and Public Health Service Policy on Humane Care and Use of Laboratory Animals. All animals were housed individually on 12-h dark-light cycles (6:00 a.m.—6:00 p.m.) with regular rodent chow and water available *ad libitum* (temperature 21°C ± 2°C and relative humidity 60% ± 15%, means ± SD).

### 2.1 Telemetry implant

Male C57BL/6J mice (11 weeks old, The Jackson Lab, Sacramento, CA) were anesthetized with isoflurane (5% induction, 1%–3% maintenance). The criteria for adequacy of anesthesia include 1) no eye blink reflex, 2) no paw pinch withdrawal, 3) no whisker movement, and 4) no irregular or sudden changes in breathing frequency. A pressure + biopotential telemetry device (HD-X11, Data Sciences International, St. Paul, MN, United States) was implanted subcutaneously in the left side of the body via a small midline incision at the ventral neck region. The pressure catheter tip was placed in the aortic arch through the left carotid artery, and the biopotential leads were tunneled subcutaneously. The negative lead was secured to the upper right pectoral muscle wall, and the positive lead was sutured just medial of the xiphoid process for recording the electrocardiogram (ECG) in the lead II configuration. Mice were given buprenex (0.05 mg/kg) subcutaneously prior to surgery and twice daily for 2 days after surgery to manage post-op pain.

### 2.2 SHS exposure

Two weeks after the telemetry implantation, mice were randomly assigned to either SHS exposed (n = 18) or filtered air (FA, n = 20) control group. SHS exposure was comparable to that of a “smoky bar” (3 mg/m^3^ of total suspended particulate [TSP]) (Semple et al., 2007; Pacheco et al., 2012) for 6 hours per day (9 a.m.–3 p.m., during light cycle) and 5 days per week (Monday through Friday). Animals in the SHS group were exposed to SHS for 12 weeks, followed by 4 weeks of SHS cessation (‘recovery’ from smoke) to model the effects of a smoke-free environment following SHS exposure.

3R4F cigarettes, an international standard reference cigarette for smoke research (Hamad et al., 2017), from the University of Kentucky Tobacco and Health Research Institute (Lexington, KY, United States) were used to generate sidestream cigarette smoke as a surrogate for SHS (Chen et al., 2008; Sekizawa et al., 2008; Wang et al., 2018; Sun et al., 2021). Two cigarettes at a time were smoked in a staggered fashion under Federal Trade Commission conditions at a rate of 1 puff/min (35 mL/puff, 2 s duration). The smoke was diluted to the target concentration with FA in a mixing chamber and then passed into a 0.44 m^3^ stainless steel-and-glass Hinners-type exposure chamber. Air sample from the outlet of the exposure chamber was collected for 15 min during the exposure period every day to determine nicotine concentration in the air with gas chromatography. Mice were exposed (whole-body exposure) in their home cage with wire lids and were provided with regular rodent chow and water *ad libitum*. TSP concentration was sampled gravimetrically taken in the morning and again in the afternoon daily. Carbon monoxide concentration was measured every 30 min during the exposure period using a carbon monoxide analyzer (X-STREAM Gas Analyzer, Rosemount Analytical, Orrville, OH). The SHS exposure condition was TSP 3.0 ± 0.2 mg/m^3^, nicotine 0.2 ± 0.1 mg/m^3^, and carbon monoxide 15.6 ± 1.8 ppm (means ± SD).

### 2.3 sEH inhibitor treatment

In separate groups of mice, an sEH inhibitor, 1-trifluoromethoxyphenyl-3-(1-propionylpiperidin-4-yl) urea (TPPU), was added to the drinking water (15 mg/L) during the 12 weeks of FA/SHS exposure period. This TPPU concentration in drinking water has been shown to reduce neuroinflammation (Ghosh et al., 2020), inflammation associated with myocardial infarction (Sirish et al., 2020), and an inflammation model of depression (Ren et al., 2016). Since TPPU is a high melting and lipophilic crystal, it was added to drinking water in a true solution of PEG 400 to give a final concentration of PEG of under 1%. The water intake for C57BL/6J mice is ∼0.25 mL/g/day (Bachmanov et al., 2002) and the estimated TPPU dose was 3.75 μg/g/day. Mice were randomly assigned to one the of the following three groups: 1) FA exposure + regular drinking water (FA-H_2_O, n = 12); 2) FA exposure + TPPU in drinking water (FA-TPPU, n = 12); and 3) SHS exposure + TPPU in drinking water (SHS-TPPU, n = 22).

### 2.4 Recording protocol

BP and ECG signals were recorded continuously for 36 h every month when mice were not exposed (Friday night to Sunday morning). Recordings were performed in a dedicated animal housing room in which no personnel entered or disturbed animals during the recording period. ECG was recorded at 4 kHz and BP was recorded at 500Hz with Ponemah software (Data Sciences International). Data were stratified by 12-h circadian window: The dark cycle (dark 1) immediately after Friday’s exposure and the following light and dark (dark 2) cycles.

### 2.5 Stress protocol

On Sunday morning, 2 hours into the light cycle (which served as the “baseline” for the stress response), mice were placed in a clear, plastic restrainer for 2 hours (“restraint” period). Mice were freed from restraints after this window, and an additional 6 hours of BP and ECG data were recorded.

### 2.6 Generating time-domain HRV measures

ECG R-waves were marked using the Analysis Attributes feature of Ponemah (Data Sciences International): 25% for QRS detection threshold; 0.03–0.25 mV for minimum R deflection; 1,500 bpm for maximum heart rate; 400 bpm minimum heart rate; and 20% peak bias. All R-R intervals longer than 400 ms were excluded. In addition, any R-R intervals that differed from either adjacent RR intervals by more than 20% were excluded using Data Insights software. This 20% change exclusion criterion has been shown to correctly identify nearly all normal-to-normal R-R intervals without compromising the specificity of excluding abnormal R-R intervals for reliable time-domain HRV analysis in rodents (Karey et al., 2019). Standard time-domain HRV measures (Malik et al., 1996) were calculated for each 12-h period.

### 2.7 Assessing baroreflex function

Baroreflex sensitivity (BRS) was evaluated with the sequence method (Bertinieri et al., 1985; Horwitz et al., 2013) using Data Insights software (Data Sciences International). Spontaneous baroreflex sequences of three or more consecutive beats in which systolic BP (SBP) and R-R interval (with three beats delay) progressively rose (or decreased) were identified by the software (Laude et al., 2009). The threshold for changes in SBP was set at 0.5 mmHg and the threshold for changes in R-R interval was set at 5 ms. The slope of the linear regression on R-R interval vs. SBP was generated for each baroreflex sequence. Slopes with r^2^ values >0.85 were accepted and averaged for each 12-h period.

### 2.8 Statistical analysis

Data are expressed as means ± SEM unless otherwise indicated. All statistical analyses were performed with GraphPad Prism (GraphPad Software, San Diego, CA). For determining SHS effects, each light cycle was analyzed separately. A two-way repeated measure ANOVA was used to evaluate the effect of exposure (FA vs. SHS) and exposure time (4, 8, and 12 weeks), followed by Fisher’s LSD post-hoc tests when appropriate. A *t*-test was used to evaluate the difference between the FA- and SHS-exposed mice after 4 weeks of recovery from SHS exposure (week 16). For stress tests (weeks 4, 8, and 12), peak BP and HR responses were compared with a two-way repeated measure ANOVA (exposure x time). A *t*-test was used to analyze stress responses after 4 weeks of recovery from SHS exposure (week 16). In addition, diurnal variations (day-night differences) were calculated by subtracting measures in the light cycle from those in dark 2 cycle and analyzed the same way as described above.

For determining effects of the sEH treatment, data from FA-TPPU and SHS-TPPU groups were expressed as Δ% from the mean values of the FA-H_2_O group and analyzed with a two-way repeated measure ANOVA (exposure x time), followed by Fisher’s LSD post-hoc tests when appropriate. A *t*-test was used for comparing the difference between FA-TPPU and SHS-TPPU mice after 4 weeks of recovery from SHS exposure. *P* < 0.05 was considered statistically significant.

## 3 Results

**FIGURE 1:**
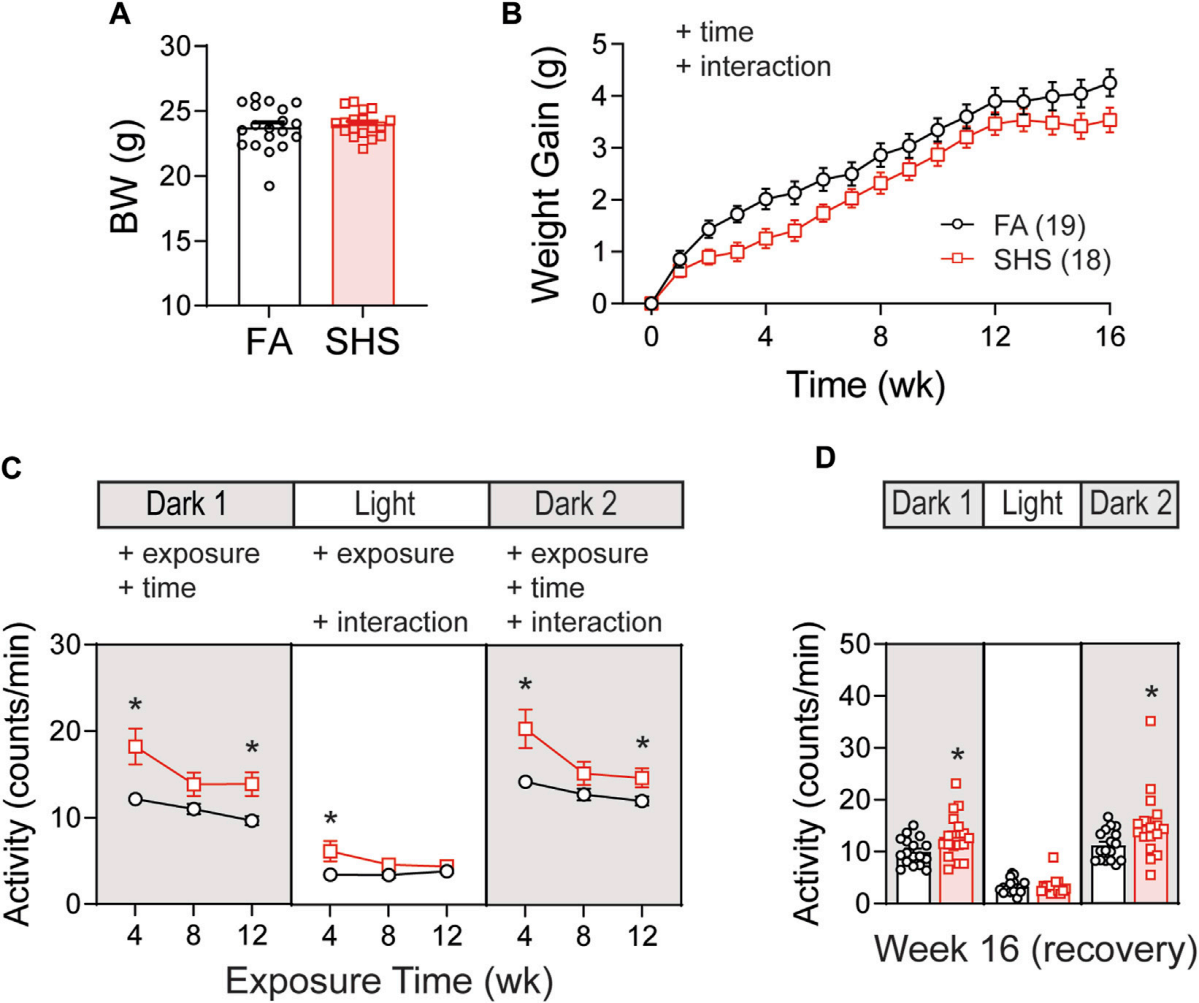
Body weight (BW) and activity over the 16 weeks of study. **(A)**. BW before exposure (week 0). There was no difference between filter air (FA)- and secondhand smoke (SHS)-exposed mice (*t*-test, *p* > 0.05). **(B)**. Weight gain over the 16 weeks. The SHS group had significantly less weight gain (two-way repeated ANOVA). **(C)**. Activity levels after 4, 8, and 12 weeks of exposure. SHS-exposed mice had higher activity levels (two-way repeated ANOVA followed by Fisher’s LSD tests). **(D)**. Activity levels after 4 weeks of cessation from SHS. The SHS-exposed mice still had higher activity level during dark cycles (*t*-test). Significant main effects from ANOVA tests are indicated with “+”. *p < 0.05, FA vs. SHS. Numbers in parentheses indicate sample sizes.

Body weights before the exposure were similar between FA (n = 19) and SHS (n = 18) groups (Figure 1A). Mice in the SHS group gained less weight over 12 weeks of SHS exposure and during 4 weeks of FA recovery (Figure 1B). The DSI telemetry system estimates the animal’s activity level based on the strength of the telemetry signal transmitted to the receiver antennas. Both the orientation of the animal relative to the receiver and the distance from the animal to the receiver antennas can change the signal strength. An activity count was generated when the signal strength changes by a software pre-defined amount and the recording system reports activity in counts per minute. Mice decreased their nighttime activity levels over the 12 weeks FA/SHS exposure period (Figure 1C). SHS-exposed mice had higher activity levels across all three light cycles (Figure 1C), an effect that was greatest after 4 weeks of exposure. The higher activity level in the SHS group persisted in dark cycles 4 weeks after cessation of SHS exposure (Week 16, Figure 1D). The higher activity level may indicate SHS-induced sleep disturbances (Morioka et al., 2018; Safa et al., 2020).

### 3.1 SHS exposure on BP and HR

**FIGURE 2:**
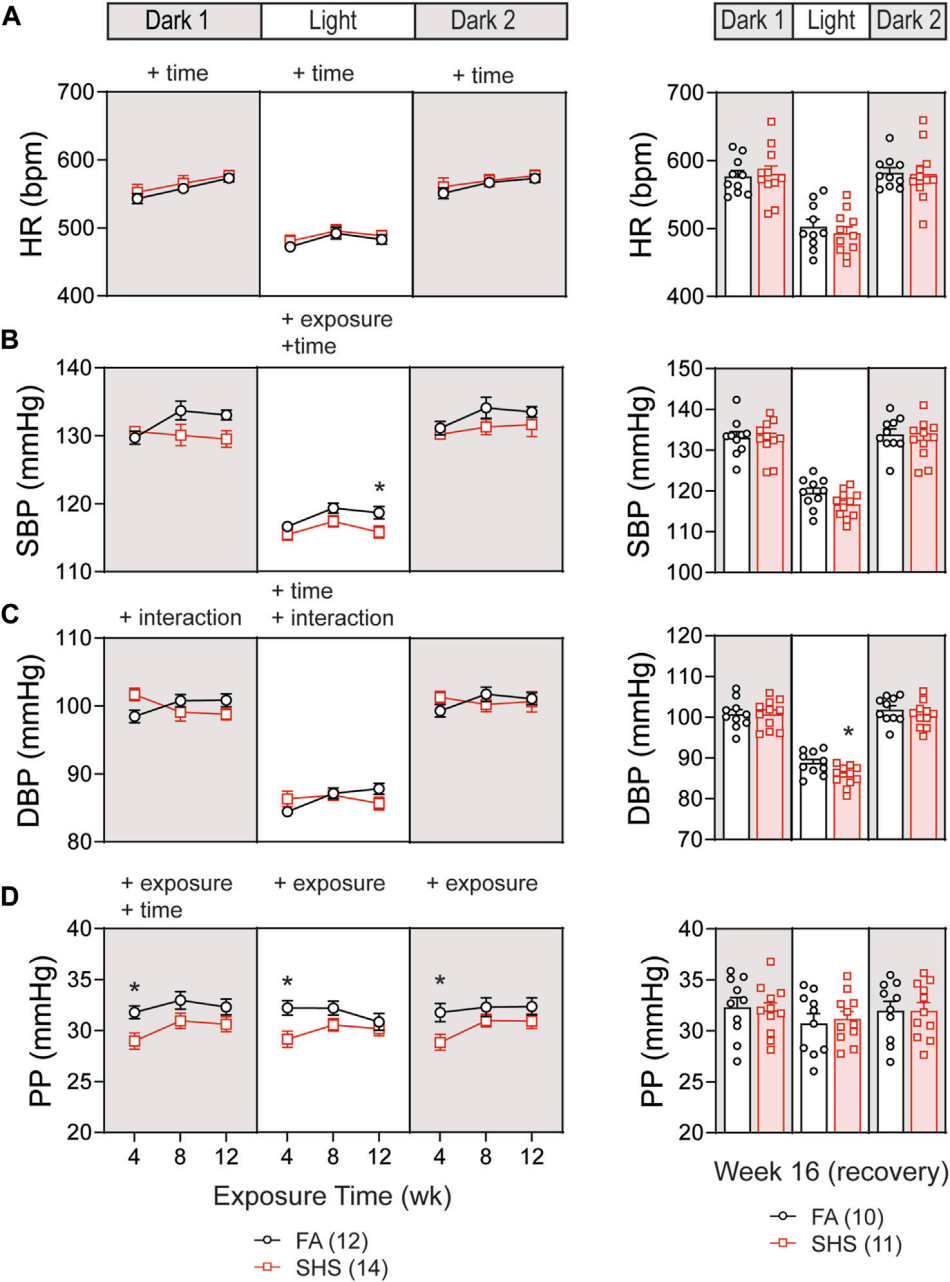
Blood pressure (BP) and heart rate (HR) over the 16 weeks of study. **(A)**. HR increased over time in both FA- and SHS-exposed groups. There was no exposure effect. **(B)**. SHS exposure lowered systolic BP (SBP) during the light cycle. **(C)**. Diastolic BP (DBP) initially was higher in the SHS group after 4 weeks of exposure but subsequently dropped to below those of FA. The SHS group had significantly lower DBP in the light cycle 4 weeks after recovery from SHS exposure. **(D)**. Pulse pressure (PP) was significantly lower in the SHS group that lasted for all three light cycles but recovered after the 4-week recovery period. Significant main effects from two-way ANOVA tests are indicated with “+”. *p < 0.05, FA vs. SHS. Numbers in parentheses indicate sample sizes.

At least 12 weeks of BP signals were successfully recorded from 26 mice in the no TPPU treatment experiment (12 FA and 14 SHS mice). There was a gradual increase in HR over the 12-week FA/SHS exposure period, however, no significant SHS effect on HR was observed throughout the whole experiment period (Figure 2A). There was a significant overall exposure effect on SBP during the light cycle, with 3 mmHg lower SBP in the SHS group (compared to the FA group) after 12 weeks of exposure (Figure 2B). This SHS-induced decrease in SBP recovered after removal from SHS exposure for 4 weeks (Figure 2B, right). Diastolic BP (DBP) showed an initial elevated trend after 4 weeks of exposure (compared to the FA group) but subsequently dropped below those of FA (Figure 2C). Interestingly, DBP during the light cycle was significantly lower in the SHS group at week 16, 4 weeks after cessation of SHS exposure (3.2 mmHg lower than the FA group). Mice from the SHS-exposed group showed a lower pulse pressure (PP) across all three light cycles (Figure 2D). This SHS-induced decrease in PP was most significant after 4 weeks of exposure and recovered after the cessation of SHS exposure (Figure 2D, right). The reduced PP in the SHS-exposed mice raises the possibility of SHS-induced reduction in the cardiac function.

### 3.2 SHS exposure on HRV and BP regulation

**FIGURE 3:**
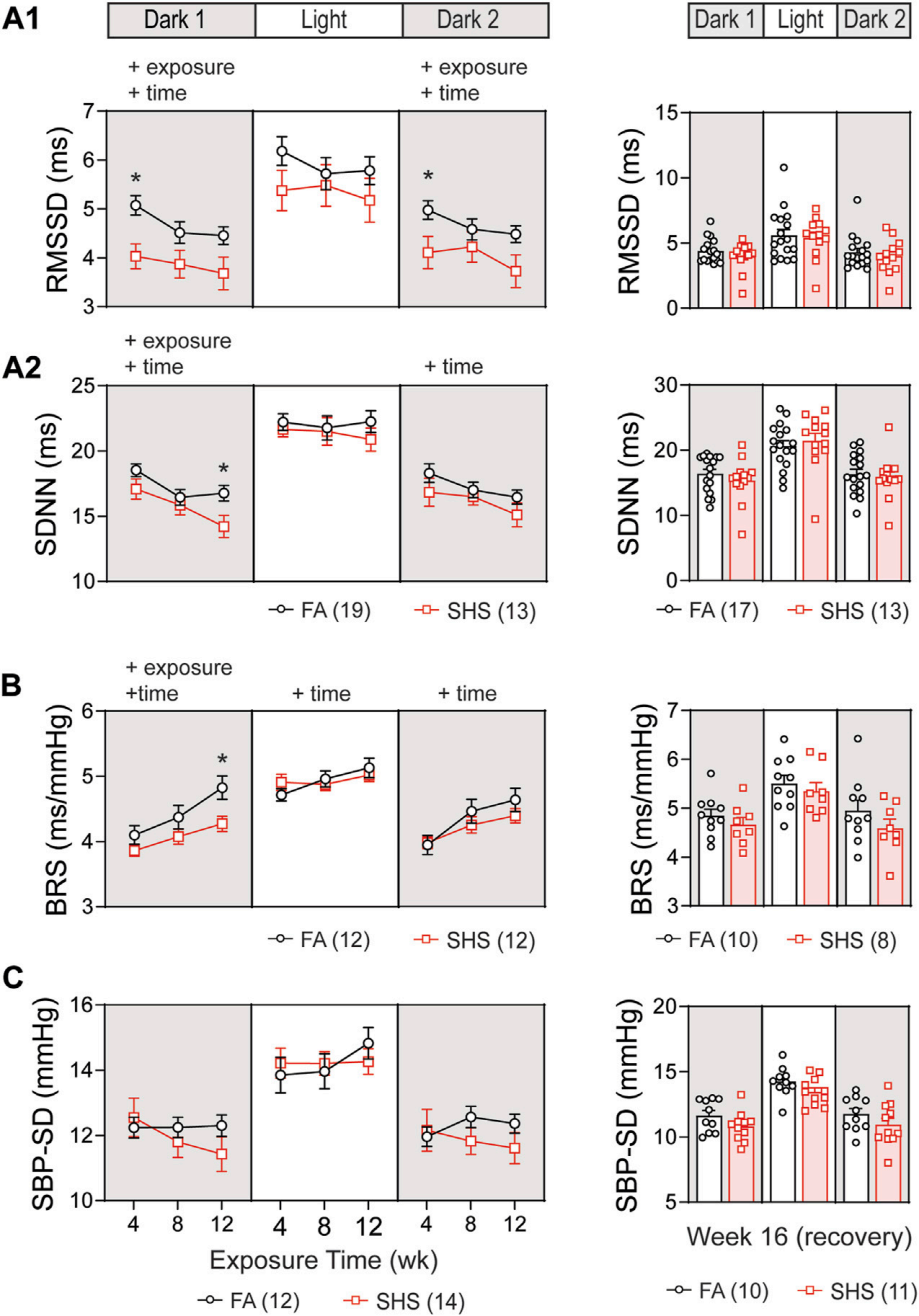
Heart rate variability (HRV) and BP regulation over the 16 weeks of study. SHS-exposed mice had significantly lower short-term **(A1)** and overall **(A2)** HRV during dark cycles. Four weeks after the cessation of SHS exposure, there was no difference in HRV between the FA- and SHS-exposed groups. **(B)**. Baroreflex sensitivity (BRS) was reduced in the SHS-exposed group only during the first dark cycle. **(C)**. There was no significant SHS exposure effect on SBP variability. RMSSD, root mean square of successive differences; SDNN, standard deviation of normal-to-normal RR intervals; SBP-SD, standard deviation of SBP. Significant main effects from two-way repeated ANOVA tests are indicated with “+”. *p < 0.05, FA vs. SHS. Numbers in parentheses indicate sample sizes.

In the no TPPU treatment experiment, 32 mice (19 FA and 13 SHS mice) had at least 12 weeks of ECG signals for obtaining measures of HRV. Short-term HRV (RMSSD, root mean square of successive difference), a measure of parasympathetic regulation, was significantly lower in the SHS-exposed group (21% lower than the FA group at week 4) during the dark cycles (Figure 3A1). The SHS effect on RMSSD recovered 4 weeks after the cessation of SHS exposure (Figure 3A1, right).

Overall HRV (SDNN, standard deviation of all normal-to-normal RR intervals), a measure of both sympathetic and parasympathetic regulation, decreased over time in both FA- and SHS-exposed groups during the dark cycles (Figure 3A2). For exposure effects, SHS significantly decreased SDNN during the first dark cycle (Figure 3A2). This reduction in SDNN was most pronounced after 12 weeks of SHS exposure (15% lower than the FA group in the first dark cycle). There was a complete recovery 4 weeks after the cessation of SHS exposure (Figure 3A2, right). A reduction in RMSSD and SDNN suggests that SHS exposure significantly altered autonomic function.

A total of 24 (12 FA and 12 SHS) mice with both BP and ECG signals were used for BRS analysis in the no TPPU treatment experiment. There was an overall exposure effect on BRS during the first dark cycle (Figure 3B). The BRS in the SHS-exposed group was 6%, 7%, and 11% lower than those of the FA control group (weeks 4, 8, and 12, respectively), suggesting a lessened BP regulation. This SHS-induced lowering of BRS did not persist beyond the first dark cycle, as no significant differences were detected during the subsequent light and second dark cycle. After the 4-week recovery from SHS window, the reduced BRS observed during SHS exposures could no longer be detected (Figure 3B, right). Despite a trend for lower HRV and BRS in the SHS-exposed group, there was no detectable difference in BP variability (standard deviation of SBP) throughout the 12 weeks of exposure (Figure 3C).

### 3.3 SHS exposure on diurnal variation and stress response

**FIGURE 4:**
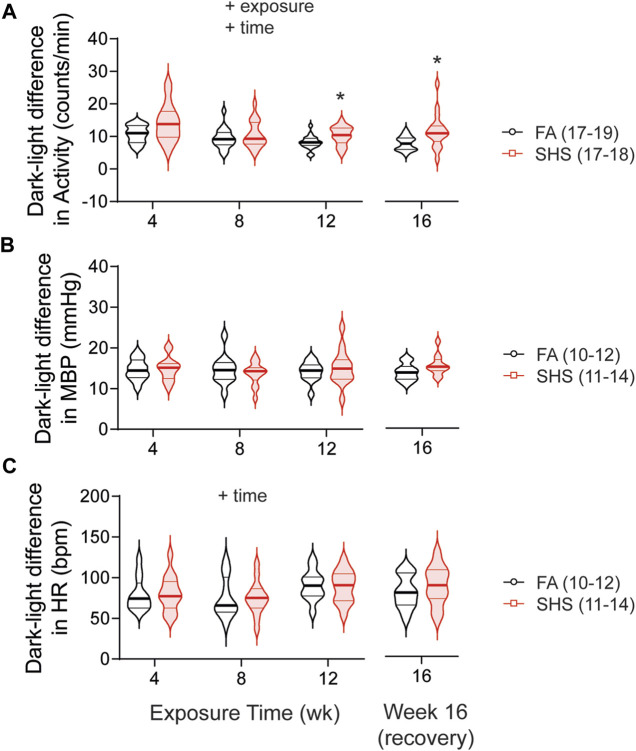
Dark-light difference (subtracting measures in the light cycle from those in dark 2 cycle) over the 16 weeks of study. SHS-exposed group had significantly greater diurnal variation in activity level **(A)** than the FA-exposed group, an effect that persisted 4 weeks after the cessation of SHS exposure. FA- and SHS-exposed mice had similar diurnal variation in MBP **(B)** and HR **(C)**. Significant main effects from two-way repeated ANOVA are indicated with “+”. *p < 0.05, FA vs. SHS. Numbers in parentheses indicate sample sizes.

As expected, mice were three times more active during the dark cycle compared to the light cycle. This dark-light difference in activity was significantly greater in SHS-exposed mice, an effect that persisted 4 weeks after the cessation of SHS exposure (Figure 4A). Nighttime mean BP (MBP) was ∼15 mmHg higher than daytime MBP, and this diurnal variation in MBP was similar in FA- and SHS-exposed mice (Figure 4B). Similarly, HR was ∼80 bpm higher during the dark cycle for both FA- and SHS-exposed groups (Figure 4C).

**FIGURE 5:**
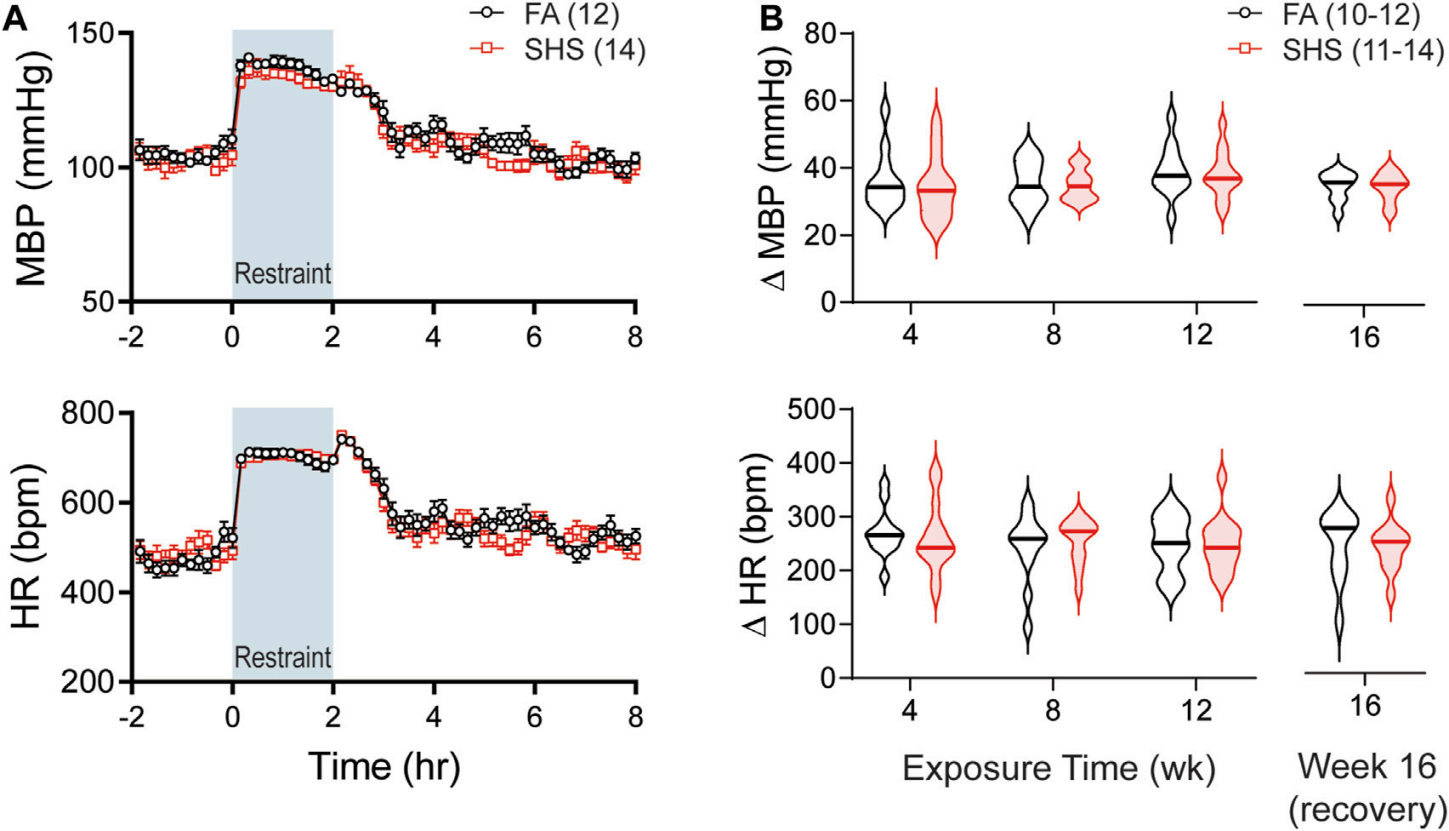
BP and HR responses to restraint stress. **(A)**. Group data of MBP and HR before, during, and after a 2-h restraint stress in mice after 12 weeks of FA or SHS exposure. **(B)**. Group data of peak pressor and tachycardic response to restraint stress over the 16 weeks of experiment. There was no exposure effect (two-way repeated ANOVA for weeks 4–12, *t*-test for week 16). Numbers in parentheses indicate sample sizes.

As shown in Figure 5A, restraint stress increased MBP and HR. SHS exposure had no effect on the magnitude of pressor and tachycardic responses to the 2-h restrain stress throughout the 16 weeks of experiment (Figure 5B).

### 3.4 sEH inhibitor on SHS-induced changes in body weight and activity level

**FIGURE 6:**
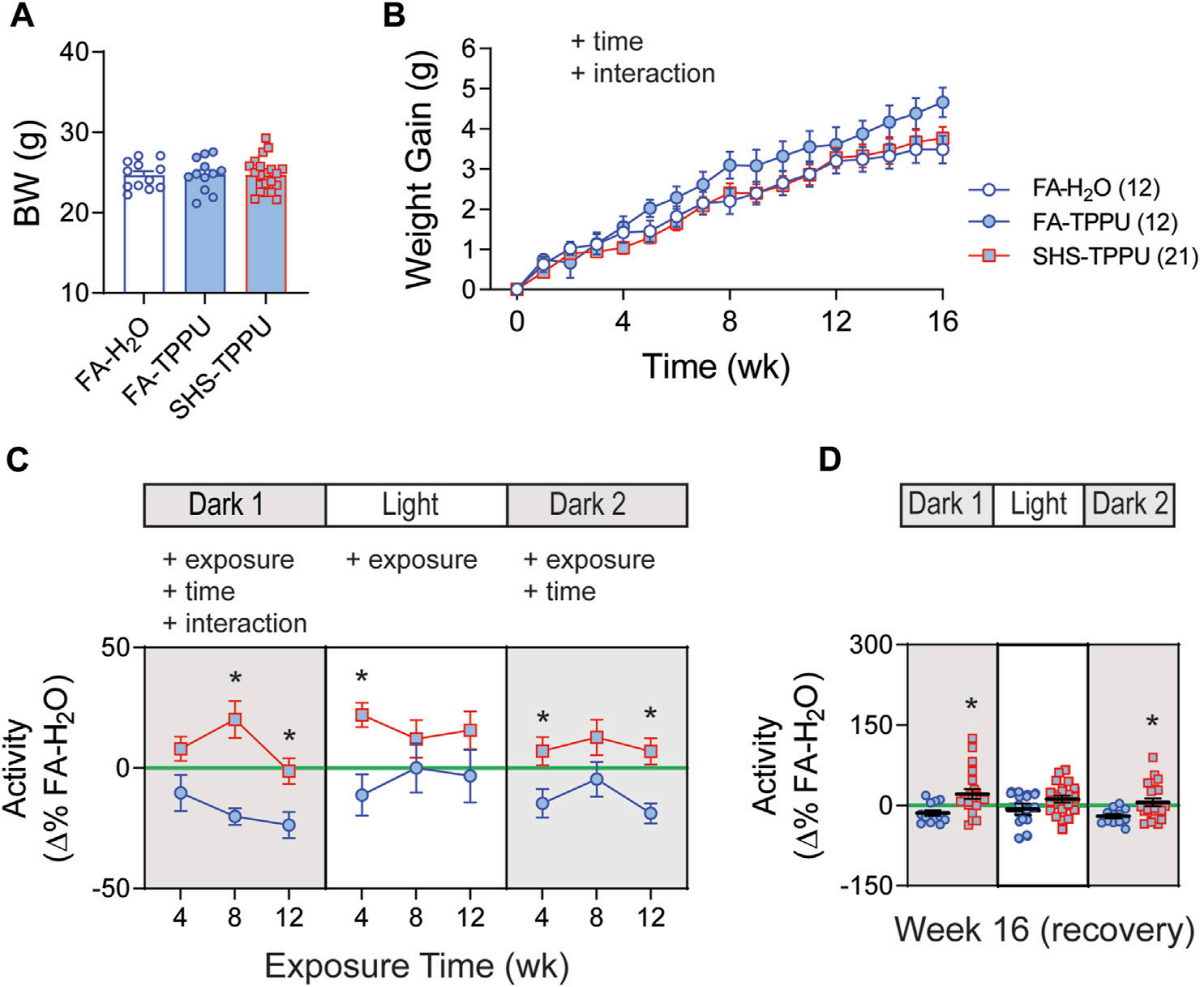
BW and activity in sEH inhibitor treatment experiment. **(A)**. BW before exposure (week 0). There was no difference among the three groups (*p* > 0.05, one-way ANOVA). **(B)**. Weight gain over the 16 weeks. FA-TPPU group had significantly greater weight gain than the FA-H_2_O and SHS-TPPU groups (two-way repeated ANOVA). **(C)**. Activity level (expressed as Δ% of the mean values from the FA-H_2_O group) after 4, 8, and 12 weeks of exposure. SHS-TPPU mice had significantly higher activity levels than those of FA-TPPU mice (two-way repeated ANOVA followed by Fisher’s LSD tests). **(D)**. Activity levels after 4 weeks of cessation from SHS exposure and TPPU treatment. The SHS-TPPU mice still had higher activity level during dark cycles (*t*-test). Significant main effects from ANOVA are indicated with “+”. *p < 0.05, FA-TPPU vs. SHS-TPPU. Numbers in parentheses indicate sample sizes.

Body weights before the exposure were similar among the three groups: FA-H_2_O, FA-TPPU, and SHS-TPPU (Figure 6A). FA-TPPU mice had greater weight gain over the 16 weeks compared to the FA-H_2_O and SHS-TPPU groups (Figure 6B). To determine the effects of the sEH inhibitor treatment on SHS-induced changes in activity levels, data from FA-TPPU and SHS-TPPU groups were expressed as Δ% from the mean values of the FA-H_2_O group (Figures 6C,D). TPPU did not prevent SHS exposure-related increase in activity levels (Figure 6C). As observed in the no TPPU treatment experiment, the higher activity levels in the SHS-TPPU group persisted in dark cycles 4 weeks after cessation of SHS exposure (Figure 6D). These data suggest that the SHS-induced increase in activity level is mediated by mechanism(s) other than inflammatory responses involving the sEH pathway.

### 3.5 sEH inhibitor on SHS-induced changes in BP and HR

**FIGURE 7:**
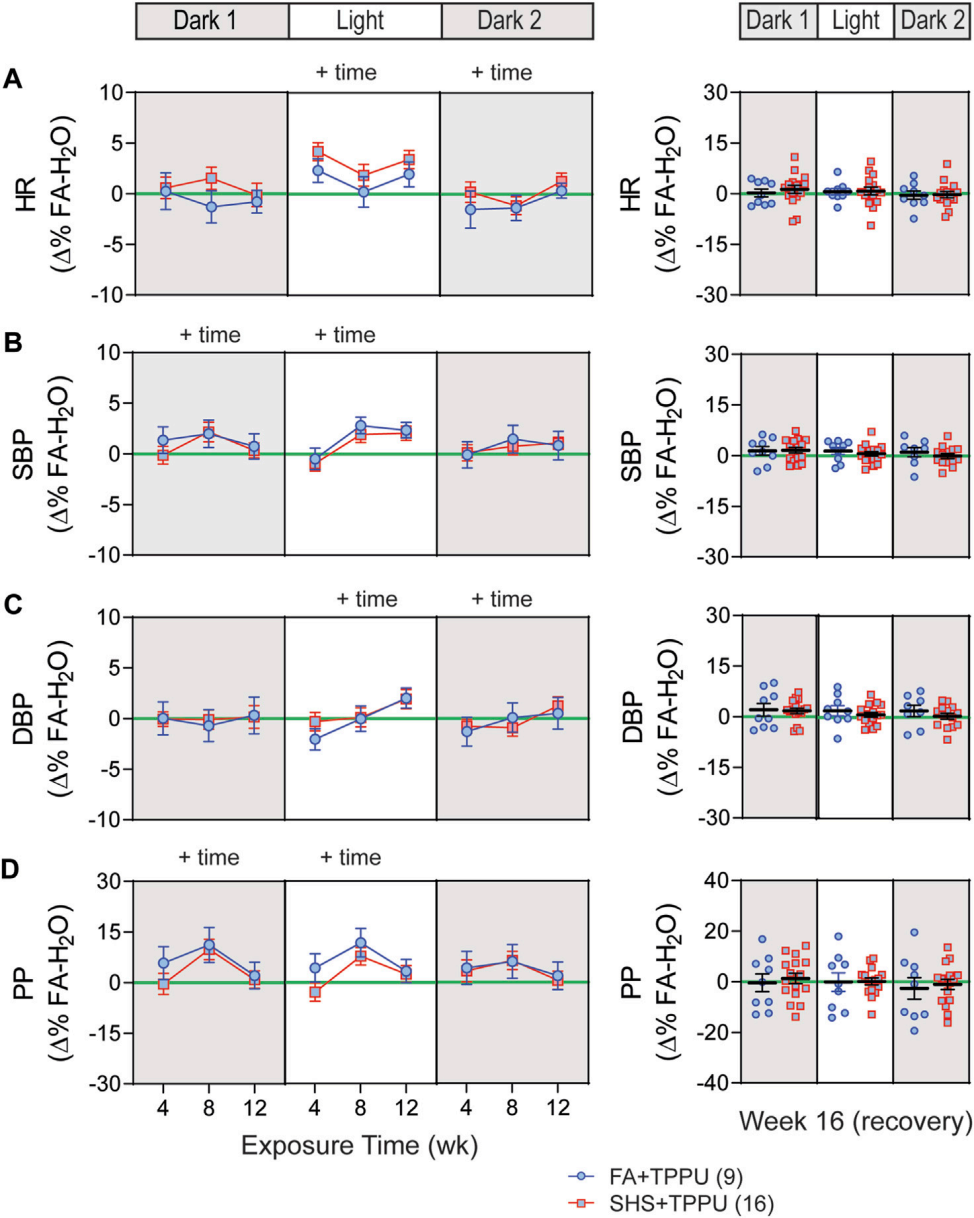
BP and HR in sEH inhibitor treatment experiment. Data were expressed as Δ% of the mean values from the FA- H_2_O group. With TPPU treatment, there was no SHS exposure effect on HR **(A)**, SBP **(B)**, DBP **(C)** and PP **(D)**. *p* > 0.05, two-way repeated ANOVA for weeks 4–12. *p* > 0.05, *t*-test for week 16 (recovery).

BP signals were successfully recorded from 33 mice in the TPPU-treatment experiment (8 FA-H_2_O, 9 FA-TPPU, and 16 SHS-TPPU mice). All data were expressed as Δ% change from the mean values of the FA-H_2_O group. As observed in the no-treatment experiment, there was no significant SHS effect on HR throughout the whole experiment period (Figure 7A). TPPU treatment eliminated the SHS-induced changes in SBP and DBP seen in the no TPPU treatment experiment (Figures 2B,C), resulting in similar SBP and DBP between FA-TPPU and SHS-TPPU groups (Figures 7B,C). TPPU treatment also prevented the SHS-induced decrease in PP (Figure 7D).

### 3.6 sEH inhibitor on SHS-induced changes in HRV and BP regulation

**FIGURE 8:**
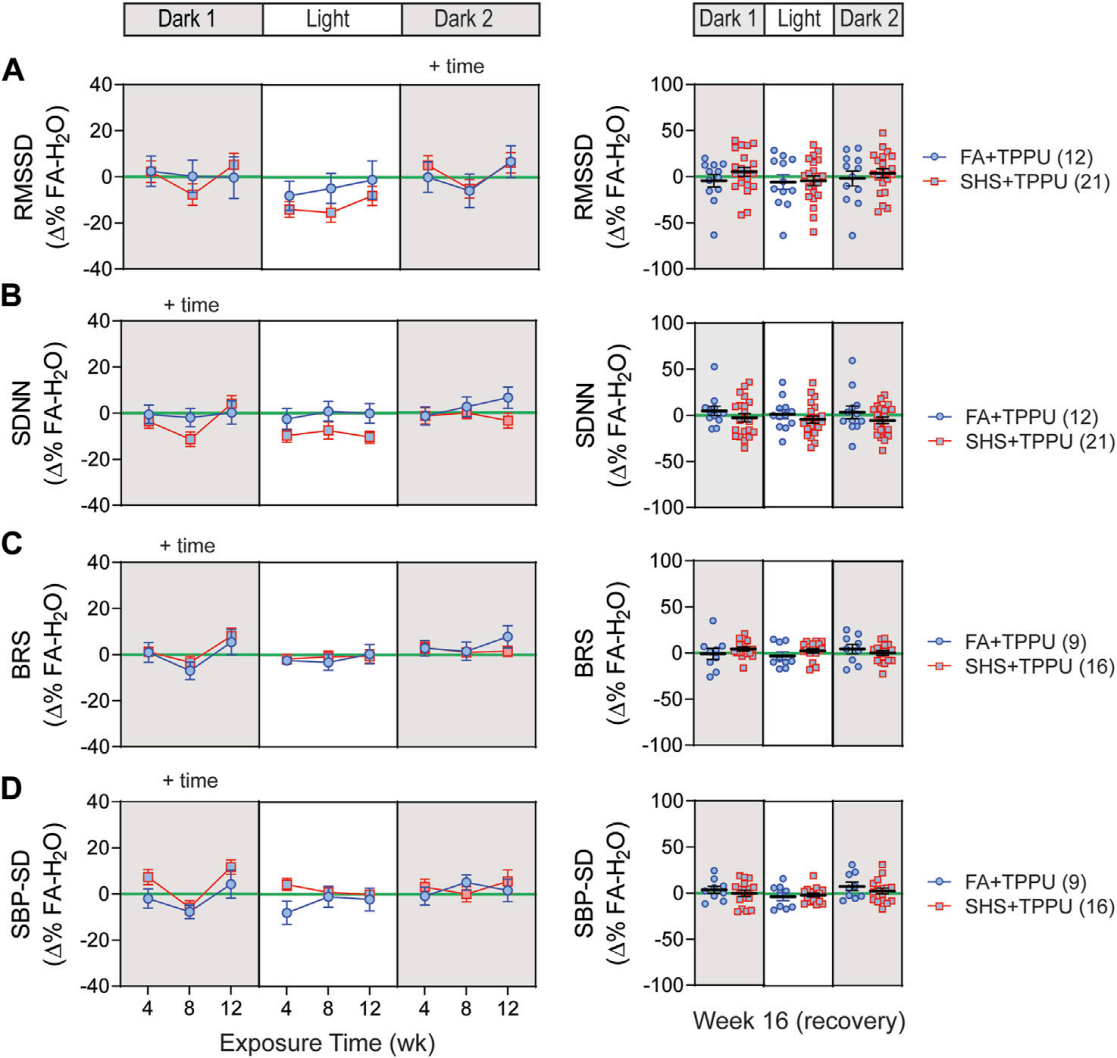
HRV and BP regulation in sEH inhibitor treatment experiment. There was no significant SHS exposure effect for short-term HRV **(A)**, overall HRV **(B)**, BRS **(C)**, and SBP variability **(D)**. RMSSD, root mean square of successive differences; SDNN, standard deviation of normal-to-normal RR intervals; BRS, baroreflex sensitivity; SBP-SD, standard deviation of SBP. Significant main effects from ANOVA are indicated with “+”. Numbers in parentheses indicate sample sizes.

ECG signals were successfully recorded from 45 (12 FA-H_2_O, 12 FA-TPPU, and 21 SHS-TPPU) mice. TPPU treatment attenuated the SHS-induced decrease in nighttime HRV seen in the no TPPU treatment experiment (Figures 3A1,A2)—there was no significant SHS effects with TPPU treatment over the 12-week exposure period (Figures 8A,B). Interestingly, there was a trend (but not significant) for lower HRV in the SHS-TPPU group during the light cycle. There was no difference in HRV after 4 weeks of the cessation from SHS exposure and TPPU treatment (Figures 8A,B).

A total of 33 (8 FA-H_2_O, 9 FA-TPPU, and 16 SHS-TPPU) mice with both BP and ECG signals were used for BRS analysis. TPPU treatment blocked the SHS exposure-induced decrease in BRS seen in the no TPPU treatment experiment (Figure 3), resulting in no difference in BRS between FA-TPPU and SHS-TPPU groups throughout the 12 weeks of exposure and 4 weeks of recovery period (Figure 8C). There was also no difference in BP variability throughout the 16 weeks (Figure 8D).

## 4 Discussion

**FIGURE 9:**
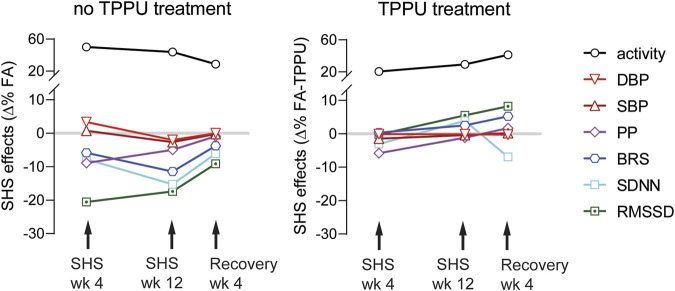
Summary of SHS exposure effects. Data points are SHS readouts expressed as Δ% of their respective FA controls. SHS’s effects on RMSSD and PP had a faster onset with maximum effect at week 4. SDNN and BRS decreased over time with maximum reduction at week 12. BP was modestly increased at week 4, followed by a modest decrease at week 12. The activity levels were higher in the SHS exposed group. Changes in cardiovascular parameters showed recovery 4 weeks after cessation of SHS. Oral TPPU treatment during prevented SHS-induced cardiovascular consequences but not activity level.

There are three main findings in this study (Figure 9): First, environmentally relevant SHS exposure (3 mg/m^3^ TSP) significantly reduced 1) two measures of HRV (RMSSD and SDNN), suggesting altered cardiac autonomic function, 2) a measure of baroreflex function (BRS), suggesting altered BP regulation, and 3) PP, suggesting a reduced cardiac function. The onset of SHS-induced attenuation of RMSSD and PP had the fastest onset, with maximum effect at week four. Conversely, SDNN and BRS decreased more gradually, reaching a maximum reduction at week 12. Second, cardiovascular consequences from SHS exposure can be reversed by removing from SHS exposure for 4 weeks. Third, oral TPPU treatment can prevent SHS-induced cardiovascular consequences. Furthermore, SHS exposure increased activity levels that persisted 4 weeks after cessation of SHS that were not prevented by TPPU treatment.

A somewhat unexpected finding of this study was the lower PP in the SHS group during the 12 weeks of exposure, an effect that recovered 4 weeks after cessation of SHS exposure. The PP is proportional to stroke volume and arterial stiffness. A lower-than-normal PP often results from a reduced stroke volume (reduced cardiac function) such as in heart failure (Schillaci et al., 2004; Yildiran et al., 2010; Petrie et al., 2014), while an abnormally high PP often comes from arterial stiffness such as in hypertension (Benetos et al., 2002). It is well-documented that SHS exposure increases arterial stiffness (Al-Dissi and Weber, 2011; Chen et al., 2015; Wang and Hu, 2015; Le et al., 2021) and decreases cardiac contractile function (de Paiva et al., 2003; Al-Dissi and Weber, 2011; Wu et al., 2014). In nonsmokers and never smokers, higher levels of serum cotinine were positively associated with higher brachial-ankle pulse wave velocity (an index of arterial stiffness) and brachial PP (Wang and Hu, 2015). Similarly, SHS significantly increased SBP, DBP, and PP in pigs (Weber et al., 2011). In contrast, in human with hypertension, SHS did not change PP despite a higher index for arterial stiffness, suggesting a counteraction from a reduced cardiac function (Gac et al., 2015). Thus, the lower PP in the SHS group seen in this study may suggest a significant reduction in cardiac function. In this regard, it is conceivable that SHS-induced compromised cardiac function may explain a small and inconsistent effect of SHS on BP.

Our results showed that 12 weeks of SHS exposure significantly reduced BRS. Prior studies showed that 3 weeks of SHS exposure had a modest, but not statistically significant, decrease in baroreflex gain assessed by infusion of vasoactive drugs in rats (Valenti et al., 2010; Valenti et al., 2011). These data suggest that the effect of SHS on baroreflex function developed over time. A reduced BRS has been shown to be associated with increased cardiovascular morbidity and mortality, including increased risks for end-organ damage and progression/development of cardiovascular disease (La Rovere et al., 2008). It has been proposed that a blunted BRS may contribute to a more delayed sustained sympathetic activation (Middlekauff et al., 2014). The sustained sympathetic activation with more chronic exposure may contribute to myocardial electrical remodeling of increased susceptibility to cardiac alternans and modification of intracellular calcium handling, known precursors to ventricular arrhythmia (Wang et al., 2018). Our results provide evidence that chronic exposure to SHS, at an environmentally relevant concentration, altered the baroreflex function that could contribute to SHS-related cardiovascular morbidity and mortality.

In general, the short-term HRV (RMSSD) reflects alterations in autonomic tone that are predominantly vagally mediated and the overall HRV (SDNN) reflects changes in both sympathetic and vagal inputs (Malik et al., 1996). Our data reveal a maximal reduction in parasympathetic regulation (RMSSD) after just 4 weeks of exposure and the measure of parasympathetic + sympathetic regulation (SDNN) showed an accumulative pattern with maximal effect after 12 weeks of exposure, suggesting that effects of SHS on sympathetic nervous system may have a slower onset and the effect is accumulative over time. Furthermore, the reduced RMSSD lasted for at least 36 h after the exposure while SDNN was only reduced in the first 12 h after the exposure (Figures 3A1, 3A2). Together, these data suggest that the parasympathetic nervous system may be more sensitive to SHS exposure with a faster onset and that continued exposure may further tip the balance of autonomic regulation towards more permanent dysfunction through activation of the sympathetic nervous system.

The parasympathetic regulation of the heart is mediated by innervations from the cardiac vagal neurons located in the nucleus ambiguous (Corbett et al., 1999; Pham et al., 2009; Sun et al., 2021). Our previous study showed that 4 weeks of SHS exposure, at the same concentration as the present study, resulted in a decreased neuronal input-output relationship of these cardiac vagal neurons (Sun et al., 2021). The reduced neuronal output is due to a higher voltage/current threshold required for action potential generation and lower spiking responses to depolarizing stimuli (Sun et al., 2021). This SHS exposure-induced decrease in neuronal output may be a general feature of particulate matter pollution as exposures to particulate matters in the form of iron-soot also decreased HRV and neuronal outputs of these cardiac vagal neurons (Pham et al., 2009).

The most significant effect of SHS on HRV attenuation occurred during the dark cycle (nighttime), when mice are generally more active. This suggests a circadian-specific effect of SHS given that SHS did not augment HR, irrespective of SHS duration or time of day. While our study models a passive smoking environment, similar circadian effects were observed in humans who smoked cigarettes, where active smoking reduced HRV—but most significantly during daytime (when humans, as a diurnal species, are typically most active). In these same individuals, HR was also not altered by smoking (Eryonucu et al., 2000).

Four weeks after removal from SHS exposure, all SHS-induced changes in BP, PP, BRS, and HRV returned to normal. These results further underscore the importance of implementing smoke-free policies. Growing evidence showed that the implementation of smoke-free laws resulted in a reduction in hospital admissions for acute myocardial infarction, coronary syndrome, and heart attack (Juster et al., 2007; Pell et al., 2008; Herman and Walsh, 2011). In 2003, New York State implemented a statewide comprehensive smoke-free law to restrict smoking in workplaces, bars, and restaurants. It has been shown that, upon the implementation of the smoke-free policy, there was a 50% decrease in the population exposed to SHS and a reduction in hospital admissions for acute myocardial infarction by 8% (Juster et al., 2007). Similarly, Herman and Walsh showed that the implementation of smoking ban in the State of Arizona resulted in a significant reduction in hospital admissions for acute myocardial infarction, stroke, asthma, and angina (Herman and Walsh, 2011). Thus, a smoke-free environment not only helps prevent non-smokers from being exposed to harmful SHS, but also helps improve the health of those who were previously exposed to SHS.

It is well recognized that SHS exposure increased lung epithelial cell permeability, increased release of proinflammatory cytokines and chemokines, and enhanced recruitment of macrophages and neutrophils (Strzelak et al., 2018). Inflammation initiated from the lungs in response to tobacco smoke results in the release of soluble substances that can trigger systemic and vascular inflammatory responses (Barnoya and Glantz, 2005; Raupach et al., 2006; Zhang et al., 2013; Raghuveer et al., 2016). In rodent animal models, tobacco smoke has been shown to induce pulmonary inflammation, increase infiltrated white blood cells recovered by bronchoalveolar lavage (Smith et al., 2005; Hartney et al., 2012) and increase pro-inflammatory cytokines (Wang et al., 2012; Strzelak et al., 2018).

Lipids such as epoxyeicosatrienoic acids (EETs) regulate important biological processes, including inflammation and immune cell behavior (Imig and Hammock, 2009; Yang et al., 2011; Pillarisetti and Khanna, 2012; Wang et al., 2013). Soluble epoxide hydrolase (sEH) catalyzes the metabolism of anti-inflammatory epoxides (such as EETs) into pro-inflammatory diols (such as dihydroxyeicosatrieneoic acids [DHETs]) (Yang et al., 2011; Pillarisetti and Khanna, 2012; Wang et al., 2013). It has been shown that exposure to tobacco smoke significantly shifted lipid mediators from anti-inflammatory epoxides to proinflammatory diols both in the lungs and plasma (Smith et al., 2005; Wang et al., 2012). Inhibiting the conversion of epoxides to diols with sEH inhibitors significantly attenuated tobacco smoke exposure-induced lung inflammatory responses (Smith et al., 2005), production of pro-inflammatory cytokines (Hartney et al., 2012), and the shift in epoxide to diol ratios (i.e., attenuated the exposure-induced decrease in epoxide-to-diol ratio) (Smith et al., 2005; Wang et al., 2012).

TPPU is metabolically stable and is absorbed efficiently through drinking water (Ostermann et al., 2015). TPPU levels in the plasma, whole blood and tissues have a remarkable linear relation to TPPU concentrations in the drinking water (Ostermann et al., 2015; Ghosh et al., 2020). TPPU concentrations in drinking water also showed a dose-dependent inhibition of the sEH activity, having a dose-dependent increase in epoxide to diol ratios of fatty acids, such as linoleic acid epoxide (EpOME) to linoleic acid diol (DiHOME) ratio (Ostermann et al., 2015). At the concentration used in the present study, the steady-state TPPU concentration was ∼1,200 ng/g in the plasma and ∼200 ng/g in the brain (Ghosh et al., 2020) and has been shown to increase epoxide to diol ratios from arachidonic acid (EETs/DHETs) and linolenic acid (EpODE/DiHODE) (Sirish et al., 2020). Our study showed that oral TPPU treatment eliminated SHS exposure-induced decreases in HRV, PP, and BRS. These data suggest that activation of inflammatory pathways contributes importantly to SHS-induced autonomic dysfunction and cardiovascular dysregulation.

Our results show that SHS exposure increased the mouse’s activity level that persisted 4 weeks after cessation of SHS exposure. As mice go through sleep-wake cycles throughout the day (Soltani et al., 2019), the higher activity level may be related to SHS-induced sleep disturbances seen in humans. In a meta-analysis, Safa and others reported that SHS exposure is significant associated with short sleep duration and poor sleep quality (Safa et al., 2020). Similarly, a cross-sectional survey of high school students throughout Japan also showed higher insomnia symptoms and sleep disturbance symptoms such as insufficient sleep and short sleep duration in never smokers with SHS exposure, compared to never smokers without SHS exposure (Morioka et al., 2018). The underlying mechanism mediating the exposure-induced sleep disturbance is not well-understood but may be related to neuronal effects of nicotine in the tobacco smoke. Saint-Mleux and others showed that activation of nicotinic receptors facilitated the release of noradrenaline in the Ventrolateral Preoptic Area, resulting in inhibition of sleep-promoting neurons and activation of wake-promoting neurons through disinhibition (Saint-Mleux et al., 2004). Taken together, these data suggest that nicotine’s effects on sleep-related neurons in the Ventrolateral Preoptic Area may contribute to the higher activity levels after SHS exposure.

In summary, the present study demonstrates that SHS exposure, at an environmentally relevant concentration, significantly reduced HRV, BRS, and PP, suggesting altered autonomic, baroreflex, and cardiac contractile functions. Evidence of these exposure-induced cardiovascular consequences were reversed during the recovery period, pointing to the importance of establishing a smoke-free environment in improving public health by preventing SHS in non-smokers as well as improving health of non-smokers with prior SHS exposure. Oral TPPU treatment prevented SHS-induced cardiovascular effects, suggesting that activation of inflammatory pathways is important in SHS effects and that sEH inhibitors maybe great candidates for preventing cardiovascular consequences of SHS exposure.

## Data Availability

The raw data supporting the conclusions of this article will be made available by the authors, without undue reservation.
